# The Editor's Letter-Box

**Published:** 1886-11-20

**Authors:** 


Mrs. Arthur Cohen, 6, Holland Park, W., writes: "I am very anxious to find a home for Annie U——, whose case you will find described in the enclosed letter, and I have been advised to write to you for advice in the matter. Her case does not seem eligible for the hospitals for incurables at Putney and Clapham, to which I have written, and I should be extremely glad to know what you consider will be the best course to adopt to find some comfortable home for her."

The enclosure is from a Norfolk clergyman's wife, and states that Annie U——, a girl of very steady and industrious habits, is afflicted with fits, which vary in character, but are at times very severe. Her parents are dead, and she has no one to help her except an only brother. When she is free from the epileptic attacks she can do plain needlework very well, and has given entire satisfaction to those who have employed her on such tasks. "She is worthy of any help she can obtain."

*** Would not Miss Paget be able to help this case? —Ed. T. H.

